# Lithium isotope traces magmatic fluid in a seafloor hydrothermal system

**DOI:** 10.1038/srep13812

**Published:** 2015-09-08

**Authors:** Dan Yang, Zengqian Hou, Yue Zhao, Kejun Hou, Zhiming Yang, Shihong Tian, Qiang Fu

**Affiliations:** 1 Institute of Mineral Resources, CAGS, Beijing 100037, P. R. China; 2 Institute of Geology, CAGS, Beijing 100037, P. R. China

## Abstract

Lithium isotopic compositions of fluid inclusions and hosted gangue quartz from a giant volcanogenic massive sulfide deposit in China provide robust evidence for inputting of magmatic fluids into a Triassic submarine hydrothermal system. The δ^7^Li results vary from +4.5‰ to +13.8‰ for fluid inclusions and from +6.7‰ to +21.0‰ for the hosted gangue quartz(9 gangue quartz samples containing primary fluid inclusions). These data confirm the temperature-dependent Li isotopic fractionation between hydrothermal quartz and fluid (i.e., Δδ^7^Li_quartz-fluid_ = –8.9382 × (1000/T) + 22.22(R^2^ = 0.98; 175 °C–340 °C)), which suggests that the fluid inclusions are in equilibrium with their hosted quartz, thus allowing to determine the composition of the fluids by using δ^7^Li_quartz_ data. Accordingly, we estimate that the ore-forming fluids have a δ^7^Li range from −0.7‰ to +18.4‰ at temperatures of 175–340 °C. This δ^7^Li range, together with Li–O modeling , suggest that magmatic fluid played a significant role in the ore formation. This study demonstrates that Li isotope can be effectively used to trace magmatic fluids in a seafloor hydrothermal system and has the potential to monitor fluid mixing and ore-forming process.

The submarine hydrothermal fluids affect mass balance of the hydrosphere^1^ and form sulfide ores on the modern seafloor^2^ and their ancient analogues preserved on land^3^. However, their sources remain controversial^4^. A common view suggests that they are derived from pure seawater circulating through the hot rocks^2^,^5^. An alternative view argues that there may be admixture of magmatic fluids escaping from magma at depth^4^. This controversy reflects the fact that transitional isotope tracers (e.g., δD, δ^18^O) seldom provide a robust signature of the fluid sources. Lithium isotope has proven to be an important geochemical tracer for fluid-related processes, particularly aqueous fluids, volatiles, and metals in magma-hydrothermal systems, because of chloride-complexed behavior of lithium in aqueous fluids^6^,^7^, strong fluid mobility^8^ and the large mass fractionation^9^. Li isotopic fractionation may be used to track mineral precipitation and/or diffusion in hydrothermal system. Quartz is the most common gangue mineral trapped ore-forming fluids as inclusions for hydrothermal deposits. Its lithium isotopic system may has potential for constraining the source of fluids and quantifying the flux of fluids from distinct sources.

In this study we first report Li isotopic compositions of the Gacun Zn-Pb-Cu deposit, a giant volcanogenic massive sulfide deposit in the Yindun arc-basin system^10^,^11^, southwest China (Fig. 1). Our data suggest that magmatic fluids escaping from a rhyolitic melt play an important role in ore formation. Additionally, we demonstrate that the Li isotopic measurements can be used to monitor the mixing of fluids associated with ore formation and could help to locate the particularly mineralized horizons.

## Results

**Figure 1:**
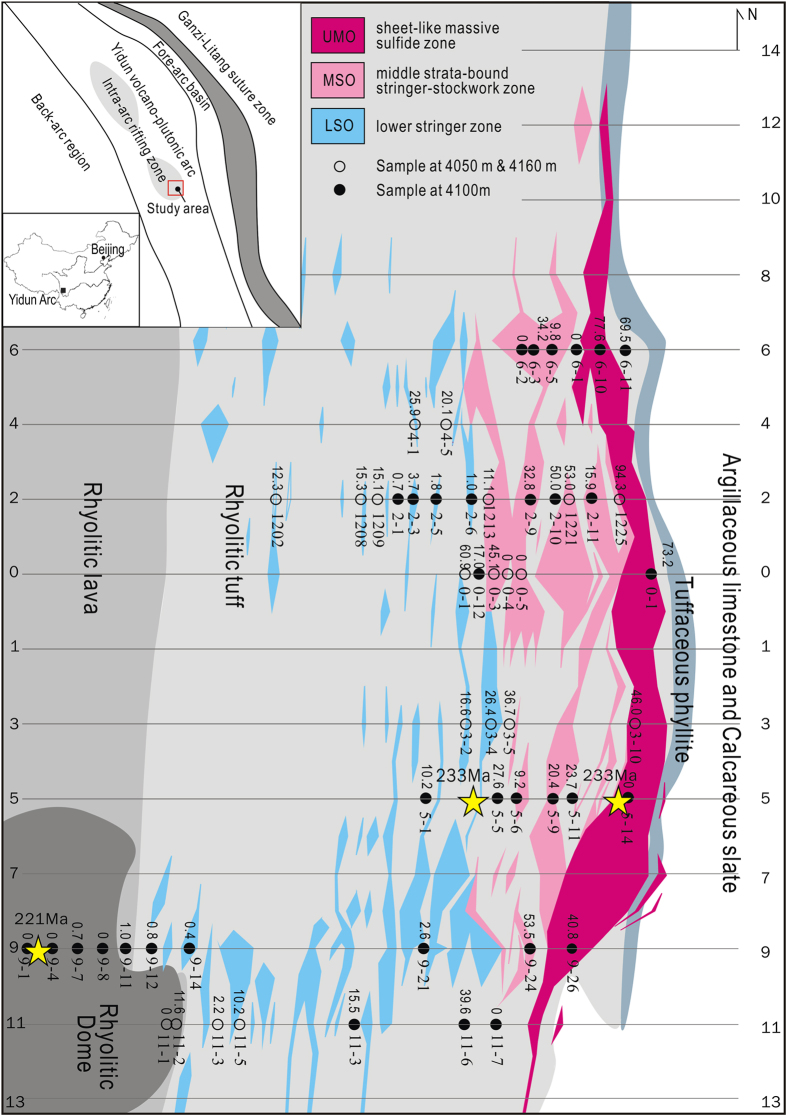
Geological map of the Gacun deposit (modified from refs. 10,12), showing sample locations and spatial distribution of the major orebodies on a 4100 m exploration plane. The Triassic volcanic-sedimentary strata were folded and steeply-dipped eastwards due to later deformation, providing an ideal cross-section that shows a rhyolitic package and the hosted orebodies from the deep (west) to top (east). Inset map shows tectonic framework of the Yidun Arc, formed by westward subduction of the Ganzi-Litang oceanic lithposhere in Triassic^10^. The Gacun deposit is located in an intra-arc rifting zone within the Traissic arc, and consists of three major orebodies. The location of U-Pb dating samples and the boundaries of a feeder zone of hydrothermal system^10^ are also shown. 53 samples for Li analyses were collected along 8 prospecting lines across orebodies at different heights (4050 m, 4100 m, 4150 m) and are shown on an exploration plane at 4100 m. Locations of all samples are marked by solid circles (at 4100 m) and open circles (at 4050 m and 4160 m). The minimal amount of seawater (X_seawater_) in the ore-forming fluids for each sample was estimated by binary mixing modeling on Li-O isotopic data of 53 quartz samples (see Appendix II, Table S1, and Fig. 3). All data from Table S1.

We have assessed a submarine hydrothermal system by a study of Li isotope on quartz and hosted fluid inclusions from the Gacun deposit, formed by Triassic submarine hydrothermal fluids^10^ (Fig. 1). This deposit is hosted by a 500-m thick, steeply-dipped, rhyolitic volcanic package exposed at Gacun (Fig. 1). It comprises three mineralized zones: (1) a sheet-like massive sulfide zone (UMO) with associated exhalites (barite, chert, jasper) covered by Triassic black phyllite, (2) a discordantly-underlying middle strata-bound stockwork zone (MSO) within a ~233 Ma rhyolitic tuff unit, and (3) a semi-layered lower stringer zone (LSO) partly hosted in a ~221 Ma rhyolitic dome^12^. These three ore zones were formed by hydrothermal fluids discharged through a sub-seafloor feeder zone rooted into a rhyolitic dome^10^,^13^ (Fig. 1). The ~233 Ma rhyolitic volcanic rocks were derived from a single, relatively shallow, differentiating felsic magma^14^. The ~221 Ma rhyolitic dome, the lastest intrusive phase, acted as a “thermal engine”, driving the circulating of fluids through the overlying volcanic package^12^.

Nine rhyolite samples were collected for Li isotope analysis in this study (Table S1). The fresh rhyolites (n = 3) outside the Gacun deposit yield a narrow δ^7^Li range from +1.0‰ to +2.3‰, similar to continental crust (δ^7^Li = +1.2 ~ +1.7‰)^15^. The alterated rhyolites (n = 6) from a feeder zone show gradually increasing in δ^7^Li values from a rhyolitic dome (+1.2‰) and the LSO host rock (+3.4‰) to MSO host rock (+8.8‰) upwards (Table S1).

**Figure 2:**
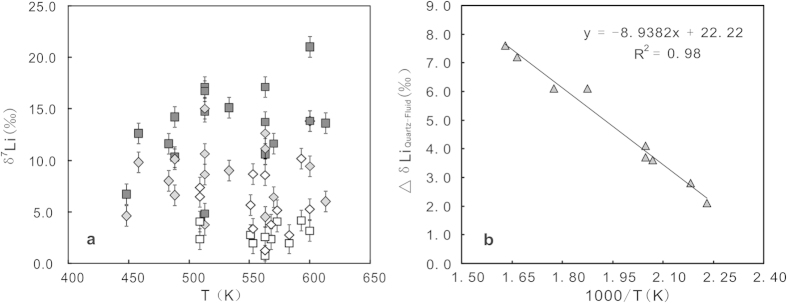
(**a**) Variation in δ^7^Li of the host quartz and fluid inclusions with the measured homogeneous temperatures. (**b**) Relationship of Li isotopic fractionation factor (△_quartz-fluid_) with homogeneous temperatures (1000/T) of fluid inclusions hosted in nine pure-quartz samples (containing primary fluid inclusions). All data from Table S1.

Fifty-three gangue quartz samples, collected across the main orebodies (Fig. 1) is divided into two groups: pure-quartz (n = 41) and quartz + sericite (n = 12). The former occurs in all three ore zones; the latter confines in the LSO and MSO. Li concentrations and isotopic compositions of 53 quartz samples and 29 fluid samples extraced from these host quartz are listed in Table S1 and plotted in Figs 2 and S1. The pure-quartz (n = 41) has Li concentrations of 0.03–1.61 μg/g and δ^7^Li varying from +4.1‰ to +22.5‰. The extracted fluids (n = 17) yield a range of δ^7^Li values from +3.7‰ to +15.0‰ (Fig. 2a). The quartz + sericite mixture has similar Li concentrations (0.02–1.75 μg/g) but lower δ^7^Li values (+0.8‰ to +4.2‰), the corresponding fluids (n = 12) have relatively high δ^7^Li varying from +1.3‰ to +10.2‰ (Fig. 2a). In general, the quartz samples from each ore zone have variable δ^7^Li values, laterally increasing outwards from a feeder zone (Table S1). The extracted fluids show average δ^7^Li values gradually increasing from +5.4‰ in the LSO to +9.8‰ in the UMO (Table S1).

All analyzed quartz samples (n = 53) were measured for O isotopic compositions, which yield δ^18^O values between +2.6‰ and +16.4‰ (Table S1). Based on homogenization temperatures (T_h_: 117°C–368°C) of fluid inclusions in analyzed quartz (Table S1), using the quation of Matsuhisa *et al*. (1979)^16^, the δ^18^O values of the fluids were estimated to vary from +0.9‰ to +8.7‰, showing a gradual decrease from a rhyolitic dome (av. +7.6‰), through the LSO (av. +7.1‰) and MSO (av. +5.4‰), to the UMO (av. +2.8‰).

## Discussion

The δ^7^Li values of both fluid inclusions and host quartz were obtained for 17 pure-quartz samples, which yield Δδ^7^Li_quartz-fluid_ values of +1.1 − +7.6‰ (Table S1). Extensive petrographic observations show that only 9 pure-quartz samples host primary fluid inclusions, evidenced by their alignment along quartz growth zones, regular forms (smooth grain, pillar, and polygon), and variable sizes (usually 3–10 μm)^10^. These 9 samples yield Δδ^7^Li_quartz-fluid_ values varying from +4.5‰ to +13.8‰ (Table S2), and show a negative correlation between Δδ^7^Li_quartz-fluid_ and 1/T (Fig. 2b), which can be described by the following linear equation: Δδ^7^Li_quartz-fluid_ = −8.9382 × (1000/T) + 22.22 (R^2 ^= 0.98; 175 °C–340 °C). The Δδ^7^Li_quartz-fluid_ shows a strong temperature- dependent fractionation.

Recent experiments on distinct mineral-fluid systems give conflicting results on Li isotopic fractionation between fluids and minerals. Lynton *et al*. (2005)^17^ studied Li isotopic fractionation in a quartz-muscovite-fluid system, and found that fluids are isotopically lighter than minerals. A study on natural samples (e.g., granitic pegmatite) also reaches a similar conclusion^18^. By contrast, an experiment on a synthetic spodumene-hydrothermal fluid system confirms a temperature-dependent fractionation, but found that fluids are isotopically heavier than coexisting spodumene^19^. This result coincides with a few empirical studies, which suggest that fluids are isotopically heavier than altered basalts at low to moderate temperatures^20^,^21^,^22^. Our result is consistent with the studies of Lynton *et al*. (2005)^17^ and Teng *et al*. (2006)^18^.

The fractionation driven by diffusion predicts that δ^7^Li difference should exist in both hosted quartz and fluid after the entrapment of fluid inclusions, and the measured results (i.e., Δδ^7^Li_quartz-fluid_) should be consistent with the traditional theory of stable isotopes: heavier isotope tending to enter into the liquid phase, due to diffusive transport in millions of years. Moreover, as this difference is often uncertain, a good correlation with the temperature is lacking. However, our data indicate that fluid inclusions were strongly lighter than their hosted quartz, and show a strong correlation between Δδ^7^Li_quartz-fluid_ and 1/T (Fig. 2b). We, therefore, argue that the result is mainly an equilibrium fractionation that occured when quartz was forming.

The empirical equation above indicates that the fluids in equilibrium with quartz have +2.1‰ to +7.6‰ lighter than their hosted quartz for these 9 pure-quartz samples. For other pure-quartz samples (n = 32) that contain secondary inclusions and failed in δ^7^Li_fluid_ measurement, their δ^7^Li_quartz_ data could be allowed to indicate the variable composition of the fluids. Based on measured T_h_ (Table S1), using above equation, we estimate their δ^7^Li_fluid_ to vary between –0.7‰ and +18.4‰ (Table S1).

It is noteworthy that the quartz + sericte group (n = 12) yields distinct Δδ^7^Li_quartz-fluid,_ varying from −6.7‰ to −0.5‰ (Table S1). The ‘δ^7^Li_quartz_’ of quartz + sericte group (n = 12) is a mixing value, due to intermingling with minor sericite. The ‘δ^7^Li_quartz_’ tends to lighter than that of pure-quartz, because δ^7^Li of micas tends to lighter^23^. However, we grinded quartz + sericte samples to >200 mesh (<44 μm) during the extraction runs for fluid inclusions, and centrifuged 30 mL leachates (distilled and deionized water: 5 rounds of 6 mL) and finally filtered using nylon filters with 0.22 μm pores (see Appendix I), which has avoided mixing of residual mineral powders. The δ^7^Li (+1.3 to +10.2‰) of the fluids extraced from quartz-sercite samples, therefore, could represent the δ^7^Li values of the ore-forming fluids at Gacun.

Our data above indicate that the Gacun ore-forming fluids are estimated to have a wider range of δ^7^Li (–0.7‰ to +18.5‰) than active vent fluids in 11°–13° EPR (+8.1‰ to +10.9‰)^20^, 21° EPR (+6.8‰ to +8.9‰)^20^, Endeavour in Judan de Fuca Ridge (+7.2‰ to +8.9‰)^21^, and Guyamas (~+10‰)^22^.

The sources of these vent fluids have been attributed to (1) progressively interacting seawater, passed through the hot rocks downward and heated by magma chamber^20^,^21^, (2) admixture of seawater-derived hot brine with cold seawater^24^, and (3) admixture of a magmatic fluid and seawater^21^. Our Li isotopic data can be used to test above hypothesis. A reaction progress model suggests that the reacted seawater gradually decrease its δ^7^Li with increasing in interaction depths and fluid temperatures, and predicts that the seawater reacted at the lowest water/rock ratios has the lightest δ^7^Li values^20^. However, this model can not explain the low-δ^7^Li signature (<4‰) of the Gacun fluids, because experiment^25^, modeling^24^, and study on natural samples^26^ indicate that the seawater reacted at <450 °C seldom yields much light δ^7^Li down to <4‰. Moreover, the lightest δ^7^Li_fluid_ values mostly cluster within or near a feeder zone at Gacun (Fig. S1), where no any organic-rich sediments were penetrated by circulating fluids (Fig. 1), and th heat transfer from a rhyolitic dome likely produced high water/rock ratios^27^, also arguing against this model. Phase separation of heated seawater could form a brine and a vapor phase^28^,^29^, however, this process did not make the δ^7^Li value decrease^21^. At Gacun, the δ^7^Li value of strongly-altered dome rhyolite (+1.2‰, GC4100-9-1, Table S1) is similar to that of the fresh rhyolites (~ + 1.2‰), which requires an extremely low δ^7^Li (≤1.2‰) hot fluid or brine interacted with the subvolcanic rock. The formation of such low-δ^7^Li brine (fluid) requires an anomalously high temperature (>500 °C)^30^ during seawater/rock interaction, which is inconsistent with microthermomatric results for fluid inclusions (Table S1). The last candidate is the injection of magmatic fluid escaping from a felsic magma, which resulted in releasing of buoyant vapor-rich fluids, localized heating, and supercritical phase separation^21^. This scenario is supported by high-temperature (>350 °C) and high-salinity (41.0 wt% NaCl) fluid inclusions hosted by phenocryst quartz in the dome rhyolite^13^, and also consistent with close spatial association of ores with rhyolitic rocks^13^, and the relatively high gas content, high salinity, and ^18^O enrichment of the Gacun hydrothermal system^10^.

**Figure 3:**
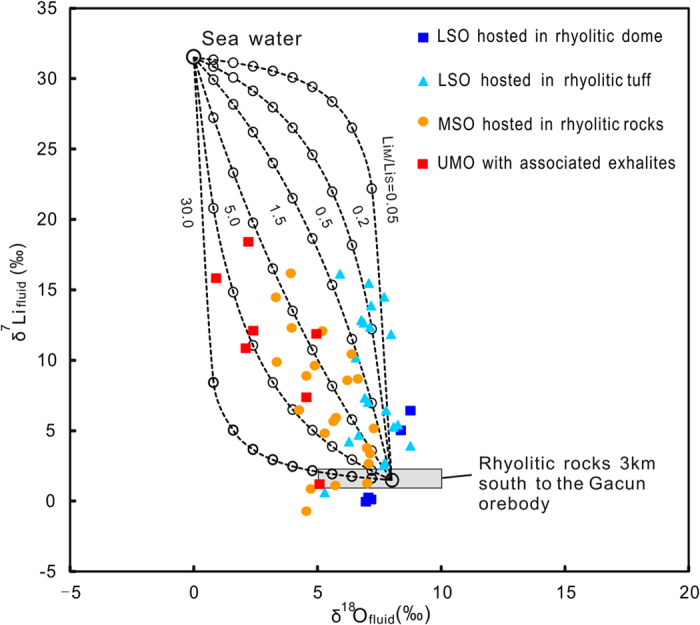
Oxygen-lithium isotopic compositions of the ore-forming fluids at Gacun, which can be reproduced by mixing of variable amounts of seawater (δ^18^O = +0‰; δ^7^Li = +31.5‰) with a magmatic fluid (δ^18^O = +8‰; δ^7^Li = +1.5‰) with variable Li_magmatic_/Li_seawater_ mass mixing ratios. Dot-lines with open-circles (10% interval) show a binary mixing between magmatic fluid and seawater. Lim/Lic refers Li concentration ratios in magmatic fluid and seawater. Modeling calculation sees Appendix II.

To date, a few experiments have been performed to determine the Li isotopic fractionation between magmatic fluids and melts^30^,^31^. A theoretic model shows that the δ^7^Li of both residual melts and exsolving fluids do not change significantly with progressive fluid exsolution^17^. Based on Li isotopic compositions of the Gacun rhyolites (δ^7^Li: +1.0‰ to +2.3‰), we estimate that the δ^7^Li values of initial fluid exsolving from a rhyolitic melt vary from +1.0‰ to +3.0, close to the lowest measured δ^7^Li values for the LSO. This means that variation in δ^7^Li_fluid_ (- 0.7 to +18.5‰) observed at Gacun may be caused by other processes, such as interacting with marine sediments (δ^7^Li = −1.0 ∼ +24‰)^32^,^33^ and mixing with seawater (δ^7^Li = +31.5‰)^24^,^26^. However, the lack of hydrothermal alteration in the hanging-wall (Triassic shales) and the absence of marine sediments in the host package at Gacun (Fig. 1) rule out the first possibility. All δ^7^Li_fluid_ data may be interpreted by a binary mixing between magmatic fluid and seawater, which is supported by Li–O isotopic system at Gacun (Fig. 3).

Figure 3 shows that δ^18^O–δ^7^Li data partially overlap with magmatic fluids, suggesting a significant role of magmatic fluids in the ore formation. However, their majority show a systemic shift toward seawater with a general negative correlation between δ^7^Li and δ^18^O for each ore zone (Fig. 3). The UMO has relatively high δ^7^Li_fluid_ (+7.4‰ to +18.4‰) and low δ^18^O_fluid_ (+0.9‰ to +4.9‰), implying more seawater contribution, whereas the LSO within the dome yields lower δ^7^Li_fluid_ (0 to +6.5‰) and higher δ^18^O_fluid_ (+6.9‰ to +8.7‰), recording the footprint of magmatic fluids (Fig. 3). Li–O mixing modeling (see Appendix II) indicates that all sample data can be reproduced by the mixing of variable amounts of seawater with a magmatic fluid. The best-fit model curves linking each ore zone can be obtained by using variable Li_magmatic_/Li_seawater_ mass mixing ratios (Fig. 3). This two-component modeling requires Li_magmatic_/Li_seawater_ mixing ratio for the UMO (1.5:1 to 5:1), much lower than those for the LSO (5:1 to 20:1). The highly variable ratios are probably controlled by the chemical compositions of the degassed magmatic fluids, which changes with the magma evolution^4^. This is because that Li partition coefficient (*D*_fluid/melt_) increases with temperature, the mole fraction of H_2_O, and the concentration of Cl in the fluids^6^,^34^. A previous study has shown that the quartz from the LSO (av. 320 °C) host CO_2_-rich multiphase inclusions, whereas ones from the UMO (av. 200 °C) host liquid-vapor two-phase aqueous inclusions at Gacun^10^. This indicates that the ore-forming fluids at Gacun vary from a mixture of CO_2_ with H_2_O to H_2_O-dominated with time, which is consistent with compositional trend produced by successively degassing of the evolving magma^4^,^35^. At similar temperatures and pressures, one can predict that relatively high CO_2_/H_2_O ratios in the fluid for the LSO likely resulted in low Li concentrations in the magmatic fluid, whereas higher concentrations of H_2_O and Cl in the fluid for the UMO probably lead to stronger Li partitioning into the fluid (Fig. 3). Assuming that a magmatic fluid has δ^7^Li of +3‰ and δ^18^O of +8‰^36^, and Triassic seawater is same as modern seawater (+31.5‰), about 80–90% and <50% magmatic fluids are estimated to account for the formation of the LSO and UMO, respectively (Table S1). Considering possible lighter δ^7^Li (~ + 26‰) of Mesozoic seawater than modern seawater^37^,^38^, our estimated results are the maxima quantitative proportions of magmatic fluids in the ore-forming hydrothermal system at Gacun.

**Figure 4:**
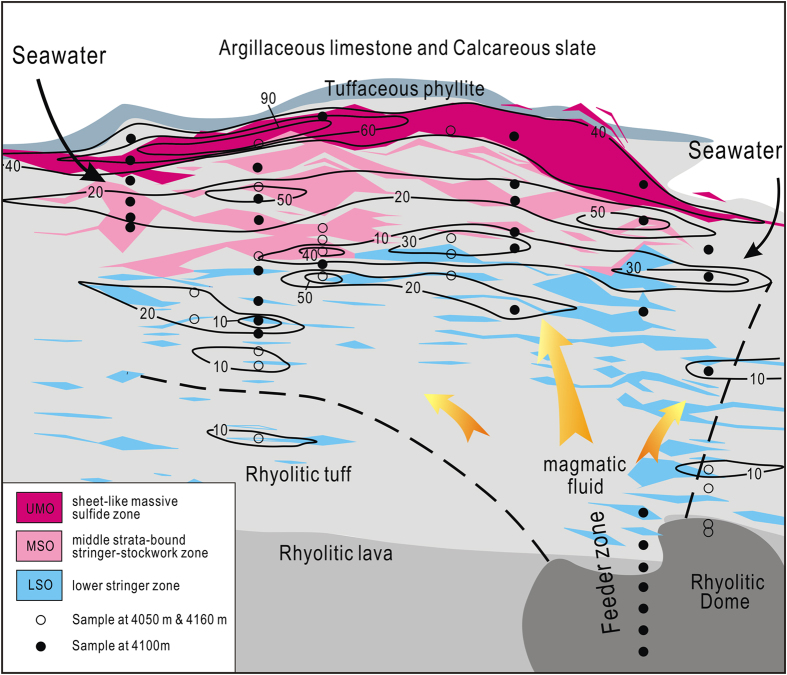
Two-dimentional compositional variation of a Triassic submarine hydrothermal system on a geological cross-section at Gacun. The map shows that the initial fluid dominated by magmatic vapors escaping from a rhyolitic dome discharged upwards via a sub-vertical feeder zone and drived the convective circulation of seawater-dominated fluids through volcanic units. The contours with tick of seawater quantitative proportion (number) for each mineralized zone were outlined, based on all point data on X_seawater_ for each sample in Fig. 1.

Spatial variation in the minimum percentages of seawater (X_seawater_) in the ore-forming fluid system shows the outline of a Triassic submarine hydrothermal system at Gacun (Figs 1 and 4), in which the convective circulation of fluids through ~233 Ma volcanic units was driven by a rhyolitic dome emplaced at ~221 Ma. The initial fluid is dominated by magmatic water, escaping from the rhyolitic dome, which firstly formed the LSO and subsequently mixed with seawater circulating through the hot rocks and discharged upward via a sub-vertical feeder zone (Fig. 4). Lateral migration of seawater mixed with magmatic fluid along permeable layers within the rhyolitic package formed the MSO orebodies (Fig. 4). The episodically inputting of fluids dominated by seawater into a seafloor brine pool within a submarine basin^10^ led to the formation of the UMO^10^,^11^.

## Conclusions

The Gacun deposit yields δ^7^Li varying from +4.5‰ to +13.8‰ for fluid inclusions and from +6.7‰ to +21.0‰ for the hosted gangue quartz(9 gangue quartz samples containing primary fluid inclusions). The estimated δ^7^Li values (−0.7‰ to +18.4‰) for the ore-forming fluids suggest a significant role of magmatic fluids in the ore formation. Our data show that combination of δ^7^Li with δ^18^O data could monitor the mixing of fluids associated with ore formation and help to locate the particularly mineralized horizons.

## Methods

### Li isotopic analysis

Li concentrations and isotopic ratios of quartz and hosted fluid inclusions were measured using an atomic absorption spectrophotometer (AAS) and a double focusing multicollector inductively coupled plasma mass spectrometer (MC–ICP–MS) at the MLR Key Laboratory of Metallogeny and Mineral Assessment, Institute of Mineral Resources, CAGS, China, following Rudnick *et al*. (2004)^39^ and Tian *et al*. (2012)^40^. The details of sample processing and analytical methods and results of microtherometric measurement and Li-O isotopic analyses are given in Appendix I and Tables S1 and S2.

Fluid inclusions were extracted by the crush and leach method at the MLR Key Laboratory of Metallogeny and Mineral Assessment, Insititute of Mineral Resources, CAGS. Before extracting fluid inclusions, approximately 4 g of handpicked quartz grains (60 ~ 80 mesh , 178 ~ 250 μm) were heated in chloroazotic acid (a ~3:1 mixture of HCl-HNO_3_) on a hot plate (T < 120 °C) for 3 hours. Then distilled-deionized water is used to clean quartz grains, until the conductivity of leachates is consistent with that of deionized water (>18.2 MΩ cm resistivity). The cleaned quartz grains were dried and grinded to be fine powders (>200 mesh, <44 μm) using an agate mortar in ultraclean cabinet. The leachates were extracted by fine powders in 30 ml of deionized water (each 6 ml, 5 times), centrifuged, and then filtered using nylon filters with 0.22 μm pores (remove silica powder). Based on traditional textural analysis of fluid inclusion populations and homogenization temperatures, primary inclusions of magmatic origin dominate the quartz samples selected for the grind-leach analysis (>90%). The fluid inclusion leachates were dried and re-dissolved in 4 M HCl, in preparation for chromatographic separation.

Dried quartz fine powders were dissolved in a mixture of concentrated HNO_3_-HF (about 1g sample in 0.5 mL HNO_3_ +5 mL HF) in savillex screw-top beakers overnight on a hot plate (T < 120 °C), followed by replenishment of the dried residua with concentrated HNO_3_ overnight and dried again, then picked up in concentrated HCl until solutions were clear. The solutions were then dried down and re-dissolved in 4M HCl, in preparation for chromatographic separation. Purification procedures with three columns were described by Tian *et al*. (2012)^40^. Lithium isotopic compositions were determined at the Institute of Mineral Resources using a standard bracketing method and a Thermo Finnigan Neptune MC-ICP-MS instrument. The δ^7^Li value of each unknown was calculated relative to the average of two bracketing IRMM-016 runs. Results of IRMM-016 (δ^7^Li = +0.2 ± 0.4‰, 2σ, n = 6) agree with previously published data (e.g., Rudnick *et al*., 2004^39^; Halama *et al*., 2007^41^, 2008^42^). During the course of this study, two international rock standards were analyzed to evaluate the accuracy of the measurements, with the basaltic BHVO-2 standard yielding a δ^7^Li value of +4.7‰ ± 1.0‰ (2σ, n = 53; Zhao *et al*., 2015^43^) and the andesitic AGV-2 standard yielding a δ^7^Li value of +6.1 ± 0.4‰ (2σ, n = 14; Zhao *et al*., 2015^43^). The external precision of Li isotopic analyses was based on 2σ values of repeat runs of pure Li standard solutions and rock solutions over a three-year period and is less than or equal ±1.0‰ (Zhao *et al*., 2015^43^). The accuracy of this method was established by Teng *et al*. (2006a)^18^ as being ±5% based on isotope dilution methods, and the precision is less than ±10% (Teng *et al*., 2006b)^44^.

### O isotopic analysis

The O isotopic compositions of quarz were analyzed at the Laboratory of Isotope, CAGS (Beijing). Single quartz grains were separated for oxygen isotope measurements. The analytical procedures of O isotopes are following Clayton and Mayeda (1963)^45^ and Hou *et al*. (2014)^46^, respectively. At ~550 °C, the quartz reacted with BrF_5_ to produce O_2_, SiF_4_ and BrF_5_ were separated from O_2_ by liquid nitrogen. At 700 °C, the O_2_ reacted with kryptol and was converted into CO_2_, which was collected in a sample tube. Oxygen isotope ratios were measured using a Thermo Finnigan MAT 253 mass spectrometer. The results are expressed in δ with V-SMOW as standards, as follows: δ^18^O_V-SMOW_ = [(^18^O/^16^O)_sample_/(^18^O/^18^O)_V-SMOW_ −1] × 1000. The analytical precisions of δ^18^O measurements were 0.2‰ (1SD), based on repeated measurements of standard samples^46^.

## Additional Information

**How to cite this article**: Yang, D. *et al*. Lithium isotope traces magmatic fluid in a seafloor hydrothermal system. *Sci. Rep*. **5**, 13812; doi: 10.1038/srep13812 (2015).

## Supplementary Material

Supplementary Information
