# Supplementary material for: Lithium isotope traces magmatic fluid in a seafloor hydrothermal system
Source: Sci Rep. 2015 Sep 8;5:13812. doi: 10.1038/srep13812 (PMC4561896; doi:10.1038/srep13812)
Supplement: Supplementary Information [file srep13812-s1.pdf]

Lithium isotope traces magmatic fluid in a seafloor hydrothermal system

Dan Yang[1], Zengqian Hou[2*], Yue Zhao[1], Kejun Hou[1], Zhiming Yang[2], Shihong Tian[1], Qiang Fu[2]

1. Institute of Mineral Resources, CAGS, Beijing 100037, P. R. China
2. Institute of Geology, CAGS, Beijing 100037, P. R. China

Supplementary Materials

APPENDIX I：METHOD FOR LI ISOTOPIC ANALYSIS

1.1 Purifying the surface of quartz

Before extracting fluid inclusions, hand-picked quartz grains (60~80 mesh , 178~250 μm) were heated in chloroazotic acid (~3:1 mixture of HCl:HNO_3) on a hot plate (T ≈ 120 °C) for 3 hours (some secondary inclusions that formed at a lower temperature are likely to have burst and been removed by this time). Distilled deionized water was then used to clean the quartz grains. To ensure that grain surfaces were completely clean, the samples were cleaned until the conductivity of leachates was consistent with that of deionized water (>18.2 MΩ) . The samples were then filtered and heated at 100 ℃ until dry. After quartz grains of different quality were processed using this method, the last soaking liquid of the quartz was analyzed by ion chromatography. Table A1 shows that, after quartz was processed using the method described above, there are no residual common ions on the grain surface.

Table A1 Ion chromatography results of the last soaking liquid from quartz

Sample number	Mineral	Sample weight (g)	Li^+	Na^+	K^+	Mg^{2+}	Ca^{2+}	F^-	Cl^-	NO_2^-	Br^-	NO_3^-	SO_4^{2-}
LY26-2	quartz	5.0210	—	—	—	—	—	—	—	—	—	—	—
		2.5078	—	—	—	—	—	—	—	—	—	—	—
		1.0187	—	—	—	—	—	—	—	—	—	—	—
		0.5198	—	—	—	—	—	—	—	—	—	—	—
		0.2567	—	—	—	—	—	—	—	—	—	—	—
		0.1120	—	—	—	—	—	—	—	—	—	—	—

Note： “—” indicates below the detection limit; all concentrations are in ng/mL.

1.2 Fluid inclusion extraction

***Bursting or grinding*：** There are two ways to open fluid inclusions: bursting or grinding. The advantage of bursting is that it is quick and easy; the main disadvantage is that some of the fluid inclusions may not burst. After fluid inclusions were extracted by the burst method, the quartz was dissolved and lithium in the residual fluid inclusions was measured along with the lithium in the quartz itself. The grinding method is time-consuming but it has the advantage that almost all of the fluid inclusions will be opened if the particle size is fine enough.

To compare the different procedures, three methods of opening fluid inclusions were tested. After surface purification, triplicate samples of ~3g each sample were weighed from the same quartz sample. Samples were either ground to 200 mesh(44 μm), ground to >200 mesh(<44 μm), or burst at 500 ℃ for 30 minutes (Yang, 2014)[1]. The same method was used to extract fluid inclusions for triplicate samples (see following Section ***Optimization of leachate extraction***). The leachates were evaporated until dry at 100 ℃ on a hot plate in a clean evaporator, and the dried sample obtained was then dissolved in 1 mL of 2% HNO_3. The resulting solution was analyzed by atomic absorption spectrometer (AAS); the results are shown in Table A2.

The results of this experiment demonstrate that the burst method (500 ℃ for 30 minutes) does not open all of the fluid inclusions (Table A2). The Li content of the obtained solution by bursting is only half of that obtained by grinding to >200 mesh (<44 μm), a loss of Li because that some fluid inclusions did not burst. The grinding method to open fluid inclusions is more thorough, and grinding to >200 mesh (<44 μm) results in few residual fluid inclusions.

The main tool that grinding method used is agate mortar. We opened fluid inclusions by grinding quartz in ultraclean cabinet. Ethanol（MOS level）was dropped into the agate mortar for avoiding loss of quartz powder. When quartz sample was ground to >200 mesh (<44 μm) , we add 1ml deionized water（18.2 MΩ）each round, a total of 6 rounds. The leachate of each round was transferred by a pipette, no ions was detected in the leachate from the fifth extraction. We had determined grinding Li blank, and no additional lithium was added during the grinding process.

Table A2 Li content of the obtained solution using three methods of opening fluid inclusions

Sample number	Sample weight (g)	Method of opening fluid inclusions	Li content of the obtained solution (ng/mL)
quartz 1	3.0085	grinding to >200 mesh, extracting 5 times, drying at 100 °C, 1 mL of 2% HNO_3	155.72
quartz 2	3.0034	grinding to 200 mesh, extracting 5 times, drying at 100 °C, 1 mL of 2% HNO_3	125.69
quartz 3	3.0077	burst at 500 °C for 30 minutes, extracting 5 times, drying at 100 °C, 1 mL of 2% HNO_3	80.32

Optimization of leachate extraction: A 3.0001 g quartz sample was ground to >200 mesh (<44 μm) and the quartz powder was ultrasonic extracted in 30 mL of leachate over five rounds (10 minutes for each round, and 6ml each round).The leachate from each round was evaporated

until dry at 100 ℃ on a hot plate in a clean evaporator, and the dried residue was dissolved in 1 mL of 2% HNO_3.

The obtained solution was analyzed by AAS; the results are shown in Table A3. The results of the leachate analyses show that there are almost no ions detected in the leachate from the fourth round, and no ions were detected in the leachate from the fifth round. Therefore, extracting leachate for five rounds will collect almost all of the ions in the liquid phase of fluid inclusions, and the content of Li in 3 g of quartz sample after grinding will meet the requirements of MC–ICP–MS analysis. The 30 mL leachate (deionized water（18.2 MΩ）: 5 rounds of 6 mL) were centrifuged and then filtered using nylon filters with 0.22 μm pores（remove the quartz powder and sericite in quartz+sericte samples）. Based on traditional textural analysis of fluid inclusion populations and homogenization temperatures, primary inclusions of magmatic origin dominate the quartz samples selected for the grind–leach analysis (>90%). The fluid inclusion leachates were dried and re-dissolved in 1 mL 4 M HCl in preparation for chromatographic separation. Purification followed the three column procedure described by Tian et al. (2012)[2] and purified samples were analyzed using a Nu-Plasma MC–ICP–MS.

Table A3 Effects of extraction time on detection results

Mineral	Extraction	Li^+	Na^+	K^+	Mg^{2+}	Ca^{2+}
quartz	1st	249.70	389.81	21.28	36.09	594.80
	2nd	132.97	213.70	5.32	5.78	148.70
	3rd	21.71	56.04	—	—	59.09
	4th	—	7.01	—	—	—
	5th	—	—	—	—	—
	6th	—	—	—	—	—

Note: "—" indicates below the detection limit; all concentration units are ng/mL.

1.3 Quartz dissolution

Ensuring that a sample is completely dissolved is critical because significant isotopic fractionation can occur between a solid and a solution[3]. The main component of quartz is SiO_2; SiO_2 dissolution in HF will generate SiF_4 (a colorless, toxic gas with an irritating odor, reaction 1).

$$SiO_2 (s) + 4HF (l) \rightarrow SiF_4 (g) + 2H_2O (l). (1)$$

One gram of quartz was dissolved in 5 mL HF. The excess HF contributes to dissolution as excess HF, and SiF_4 will form H_2SiF_6 (a strong acid, reaction 2). This is a reversible reaction: H_2SiF_6 will decompose into SiF_4 and HF, and these are removed by evaporation.

$$SiF_4 (g) + 2HF (l) \leftrightarrows H_2SiF_6 (l). (2)$$

If the sodium–magnesium–aluminum content in quartz is high, complex fluorides such as ralstonite can form during quartz digestion in HF with $HClO_4$ (ref. 4). Li can be retained in these fluorides, which cannot be easily dissolved with the addition of a small volume of $HClO_4$ (Ryan and Langmuir, 1987)[5]. Moreover, in the loading solution, such fluorides form a gel that can change the flow rate of elution and, in the worst case, can get stuck in the ion-exchange column. If the loading solution is centrifuged to remove this gel, a large proportion of lithium retained by the complex flouride will be lost. Avoiding the generation of complex fluorides (the gel) is therefore the key problem in quartz dissolution.

To avoid generating ralstonite, approximately 1 g quartz was digested in PFA Teflon screw-top beakers in a mixture of 1 mL of concentrated HNO_3 and 5.0 mL concentrated HF. After this initial step, the sample dissolution procedure followed that described by Tian et al. (2012)[2].

1.4 Sample weight

To meet the requirements of MC–ICP–MS analysis, the lithium content of the purified solution must be ≥100 ng/mL, and the lithium content of dissolved quartz must be >200 ng/mL. To investigate the lithium content of quartz in different mineralized systems, quartz vein samples from the Gacun deposit were analyzed. Quartz grains (1.0 g per sample) were hand-picked (60–80 mesh). After procedure**1.1、1.2、1.3**, the samples were added to 1 mL of 2.0% (v/v) HNO_3 solution. The lithium analysis was performed at the MLR Key Laboratory of Metallogeny and Mineral Assessment, Institute of Mineral Resources, CAGS (Beijing) China using an AAS; the results are shown in Table A4. Given the minimum lithium content of quartz in the mine, in order to meet the requirements of MC–ICP–MS analyses, the amount of sample needed for quartz from the Gacun mine is ~3g.

Table A4. Lithium concentration of quartz from Gacun deposit in China (ng/mL)

Sample	GC1202	GC1208	GC1209	GC1213	GC10-19-3
Li (ng/mL)	140	141	1304	907	62.0

1.5 Quartz major element chemistry

Table A5 shows the concentrations of major elements in quartz from the Gacun deposit. In both the Gacun volcanic-hosted massive sulfide deposit, the concentrations of major elements in the quartz veins are much lower than those in the basalt and andesite standard samples. Therefore, the lithium isotope chemical purification method described by Tian et al. (2012) [2] is also applicable to quartz samples from the Gacun deposit.

Table A5 Concentration of elements in quartz from the Gacun deposit (μg/mL)

Deposit	Number	Na	K	Mg	Ca	Cu	Fe	Zn
Gacun	GC1202	10.1	—	4.62	292	0.135	1.810	0.184
	GC1208	17.4	10.8	5.29	504	0.098	0.357	—

	GC1209	17.2	23.5	8.33	543	0.049	1.590	—
	GC1213	39.0	39.4	230	111	0.258	0.098	0.209
	GC10-19-3	24.0	15.1	6.30	215	0.025	0.357	—
international	BHVO-2	1.64%	0.43%	4.36%	8.17%	127	8.63%	103
standard	AGV-2	3.11%	2.39%	1.08%	3.72%	53.0	4.68%	86.0

Note：“—” indicates below the detection limit; all concentrations are in μg/mL. The concentrations of major elements in international standards are sourced from a sample manual provided by the United States Geological Survey

APPENDIX II：Modeling calculation

The contribution of seawater and magmatic exsolution fluids to seafloor hydrothermal ore-forming systems was modeled in this study, using the isotopic mixing principle and the δ^7Li and δ^{18}O values of seawater and magmatic water end-members. The following formulas were used to obtain the curve shown in Fig. 3:

$$\delta^7Li_{mix}= (\delta^7Li_{sea}C_{Li\text{-}sea}X_{sea}+\delta^7Li_{magma}C_{Li\text{-}magma}X_{magma}) /(C_{Li\text{-}sea}X_{sea}+C_{Li\text{-}magma}X_{magma}). \quad (1)$$

$$\delta^{18}O_{mix}= (\delta^{18}O_{sea}C_{O\text{-}sea}X_{sea}+\delta^{18}O_{magma}C_{O\text{-}magma}X_{magma}) / (C_{O\text{-}sea}X_{sea}+C_{O\text{-}magma}X_{magma}). \quad (2)$$

δ^7Li_{mix}: isotope ratio after the two sourcesare mixed;

δ^7Li_{sea}: isotope ratio of seawater, 31.5‰ (refs. 5-8);

δ^7Li_{magma}: isotope ratio of magmatic water (the highest value of fresh rhyolite in the study area is 2.3‰);

$C_{Li\text{-}sea}$: concentration of Li in seawater, 0.18ppm (refs. 6-8). $C_{Li\text{-}sea}$ is referred to as Li_s in Fig. 3;

$C_{Li\text{-}magma}$: concentration of Li in magmatic water (Li_M in Fig. 3);

X_{sea} and X_{magma}: the percentage of seawater and magmatic water, respectively, that participated in the hydrothermal system (X_{magma} = 1– X_{sea});

$\delta^{18}O_{mix}$: δ^{18}O of two mixed sources of fluid;

$\delta^{18}O_{sea}$: isotopic ratio of seawater, 0‰ (ref. 9);

$\delta^{18}O_{magma}$: isotopic ratio of magmatic water; the average δ^{18}O of magmatic water is 8.0‰ (ref. 9);

$C_{O\text{-}sea}$ and $C_{O\text{-}magma}$: concentration of oxygen in seawater and magmatic water, respectively. The approximate oxygen content of water is considered to be 88.9% for both sources.

Based on the $C_{Li\text{-}magma}/C_{Li\text{-}sea}$ of 0.05, 0.2, 0.5, 1.5, 5.0, and 30.0, we obtainthe six best-fit curves shown in Fig. 3.

Previous research suggests that there is no significant lithium isotope fractionation between melt and fluids[10-12]. Therefore, the range of δ^7Li values in magmatic water can be approximated by the δ^7Li value of relatively fresh rhyolite in the Gacun region. We consider samples with δ^7Li values of between 0 ‰ and ~2.3‰ to have not interacted with seawater (X_{sea}= 0‰).

The value of $C_{Li\text{-}magma}$ was determined according to the sample location on the $C_{Li\text{-}magma}/C_{Li\text{-}sea}$curve (Fig. 3). X_{sea} was then calculated according to equation (1) (Table S2; Fig. 3).

References

1. Yang, D., Source and evolution of ore-forming fluids in a volcanogenic massive sulfide (VMS) deposit: New constraint from lithium isotope on the genesis of the Gacun deposit, Sichuan. *PhD dissertation of China University of Geosciences* (Beijing), 47-57(2014) .

2. Tian, S.H., Hou, Z.Q. & Su, A.N. Separation and Precise Measurement of Lithium Isotopes in Three Reference Materials Using MC-ICPMS. *Acta Geologica Sinica* **86**, 1297-1305 (2012).

3. Moriguti, T., & Nakamura, E., 1998, High-yield lithium separation and the precise isotopic analysis for natural rock and aqueous samples. *Chemical Geology* **145**, 91-104 (1988).

4. Croudace, I. W. A possible error source in silicate wet chemistry caused by insoluble fluorides. *Chem Geol.* **31**, 153-155 (1980).

5. Ryan, J. G., & Langmuir, C. H. The systematics of lithium abundances in young volcanic

rocks. *Geochimica et Cosmochimica Acta* **51**, 1727-1741 (1998).

6. Chan, L. H., & Edmond, J. M. Variation of lithium isotope composition in the marine environment: A preliminary report. *Geochim Cosmochim Acta* 52, 1711-1717 (1988).

7. James, R. H., & Palmer, M. R. The lithium isotope composition of international rock standards. *Chem. Geol.* **166**, 319-326 (2000).

8. You, C.F., Castillo, P.R. & Gieskes, J.M. Trace element behavior in hydrothermal experiments: Implications for fluid processes at shallow depths in subduction zones. *Earth Planet. Sci. Lett.* **140**, 41–52 (1996).

9. Taylor, H. P. Jr & Sheppard, S. M. F. Igneous rocks: I. Processes of isotope fractionation and isotope systematic. *Rev. Mineralogy***16**, 227-272 (1986).

10. London, D., Hervig, R.L., & Morgan, G.B. Melt-vapor solubilities and elemental partitioning in peraluminous granite-pegmatite systems—experimental results with Macusani glass at 200 Mpa.*Contributions to Mineralogy and Petrology* **99**, 360–373(1988).

11. Webster, J.D., Holloway, J.R., & Hervig, R.L. Partitioning of lithophile trace-elements between H_2O and H_2O + CO_2 fluids and topaz rhyolite melt. *Economic Geology* **84**, 116–134 (1989).

12. Candela, P.A., & Piccoli, P.M. Model ore-metal partitioning from melts into vapor and vapor/brine mixtures: Mineralogical Association of Canada, Quebec, Quebec. In J.F.H. Thompson, Ed., *Granites, Fluids, and Ore Deposits* **23**, 101–128 (1995).

13. Matsuhisa,Y., Goldsmith, J. R., & Clayton, R. N. Oxygen isotope fractionation in the system quartz-albite-anorthite-water. *Geochimica et Cosmochimica Acta* **43**, 1131-1140 (1979)

Table S1 Microthermomatic data and Li-O isotopic data of the samples from the Gacun deposits in China

	Sample	Location	Sample	Mineral/ rock	Group	T_h (℃)	Li Content (μ g/g)	δ^7Li_{quartz} (‰)	δ^7Li_{fluid} (‰)	$\triangle_{quartz\text{-}fluid}$ (‰)	Estimated δ^7Li_{fluid} (‰)	$\delta^{18}O_{quartz}$ (‰)	Estimated $\delta^{18}O_{fluid}$ (‰)	$X_{seawater}$ (‰)
**LSO hosted in rhyolitic dome**														
1	GC4100-9-1	4100 m; Line 9	Fine quartz vein in silicified rhyolite	Quartz	Quartz	175-359 (240)	0.562	5.1	—	—	0.3	16.4	7.0	0.0
2	GC4100-9-7*	4100 m; Line 9	Fine quartz vein in silicified rhyolite	Quartz	Quartz	154-350 (297)	1.443	11.6	6.4	5.2	5.1	15.3	8.3	0.7
3	GC4100-9-8	4100 m; Line 9	Fine quartz vein in silicified rhyolite	Quartz	Quartz	209-356 (297)	0.152	6.7	—	—	0.2	14.1	7.1	0.0
4	GC4100-9-4*	4100 m; Line 9	Fine quartz vein in silicified rhyolite	Quartz	Quartz	240	1.611	4.8	3.7	1.1	0.0	16.3	6.9	0.0
5	GC4100-9-11	4100 m; Line 9	Fine quartz vein in silicified rhyolite	Quartz	Quartz	218-368 (320)	0.590	13.6	6.0	7.6	6.5	14.9	8.7	1.0
**LSO hosted in rhyolitic tuff**														
6	GC1202	4050 m; Line 2	Sulfide-Qtz vein in altered rhyolitic tuff	Quartz	Quartz	327	0.188	21.2	—	—	13.9	13.1	7.1	12.3
7	GC1208	4050 m; Line 2	Sulfide-Qtz vein in altered rhyolitic tuff	Quartz	Quartz	327	0.060	12.1	—	—	4.7	12.6	6.6	15.3
8	GC1209	4050 m; Line 2	Sulfide-Qtz vein in altered rhyolitic tuff	Quartz	Quartz	327	1.304	20.0	—	—	12.7	12.8	6.8	15.1
9	GC4100-2-1	4100 m; Line 2	Qtz in sericite-quartz alteration zone in rhyolitic tuff	Quartz	Quartz-Sericite	327	0.090	3.2	5.3	-2.1	5.3	14.0	8.0	0.7
10	GC4100-2-3	4100 m; Line 2	Qtz vein in silicified rhyolitic tuff	Quartz	Quartz	153-349 (327)	1.228	21.0	13.8	7.2	14.5	14.7	7.7	3.7
11	GC4100-2-5	4100 m; Line 2	Qtz veins in silicified rhyolitic tuff	Quartz	Quartz	327	0.456	9.9	—	—	2.6	13.6	7.6	1.8

12	GC4100-2-6*	4100 m; Line 2	Qtz in silicified rhyolitic tuff with disseminated sulfides	Quartz	Quartz	327	0.003	13.8	9.4	4.4	6.5	13.7	7.7	1.0
13	GC4100-0-12	4100 m; Line 2	Qtz in sericite-quartz alteration halo	Quartz	Quartz-Sericite	174-291 (215)	1.396	4.2	10.2	-6.05	10.2	12.7	6.5	17.0
14	GC4100-5-1	4100 m; Line 2	Qtz lends within rhyolitic tuff	Quartz	Quartz	117-339 (320)	0.181	14.2	10.1	4.1	7.1	13.2	7.0	10.2
15	GC4100-5-5	4100 m; Line 5	Qtz veins in rhyolitic tuff	Quartz	Quartz	156-311 (290)	0.294	22.5	—	—	16.2	13.1	5.9	27.6
16	GC4100-9-12	4100 m; Line 9	Qtz stringer within the deformed rhyolitic tuff	Quartz	Quartz	120-220 (185)	0.217	12.6	9.8	2.8	5.5	14.4	8.2	0.8
17	GC4100-9-14	4100 m; Line 9	Qtz vein in altered rhyolitic rock	Quartz	Quartz	320	1.118	11.1	—	—	4.0	14.9	8.7	0.4
18	GC4100-9-21	4100 m; Line 9	Qtz stringer in altered rhyolitic rock	Quartz	Quartz	327	1.452	19.2	—	—	11.9	13.9	7.9	2.6
19	GC4100-11-3	4100m;Line 11	Qtz in intensely-silicified rhyolitic rock	Quartz	Quartz	300	0.298	19.5	—	—	12.9	13.6	6.7	15.5
20	GC4160-4-1	4160m; Line 4	Qtz vein in rhyolitic tuff	Quartz	Quartz	284-304 (290)	1.142	10.6	4.5	6.1	4.3	13.5	6.2	23.3
21	GC4160-4-5*	4160m; Line 4	Qtz in intensely-silicified rhyolitic rock	Quartz	Quartz	290	0.061	13.7	11.1	2.6	7.4	14.1	6.9	12.7
22	GC4160-11-1	4160m; Line11	Qtz veins within rhyolitic tuff	Quartz	Quartz	260	0.417	6.1	—	—	0.6	13.7	5.3	0.0
23	GC4160-11-2	4160m; Line11	Sulfide-Qtz veins in rhyolitic tuff	Quartz	Quartz	300	0.170	22.1	—	—	15.5	13.9	7.0	11.6
24	GC4160-11-3	4160m; Line11	Sulfide-Qtz vein and stockwork in sericite-quartz alteration hlo	Quartz	Quartz-Sericite	120-340 (310)	0.690	2.0	2.8	-0.74	2.8	14.2	7.7	2.2
25	GC4160-11-5	4160m; Line11	Sulfide-Qtz veins in rhyolitic tuff	Quartz	Quartz	280	0.365	18.4	—	—	12.4	14.7	7.1	10.2
**MSO hosted in rhyolitic rocks**														
26	GC1213	4050 m; Line 2	Qtz vein in rhyolite	Quartz	Quartz	327	0.907	10.0	—	—	2.7	13	7.0	11.1

27	GC1221*	4050 m; Line 2	Sulifde-Qtz veins in rhyolite	Quartz	Quartz	240	0.032	17.1	10.6	6.5	12.3	13.3	3.9	53.0
28	GC4100-6-1	4100 m; Line 6	Qtz in sericite-quartz zone in rhyolitic tuff	Quartz	Quartz-Sericite	183-336 (290)	0.555	0.8	1.3	-0.5	1.3	14.2	7.0	0.0
29	GC4100-6-2	4100 m; Line 6	Qtz in sericite-quartz zone in rhyolitic tuff	Quartz	Quartz-Sericite	246-362 (295)	0.023	2.4	3.8	-1.4	3.8	14.0	7.0	11.1
30	GC4100-6-3	4100 m; Line 6	Qtz eyes in rhyolitic tuff	Quartz	Quartz	119-266 (210)	0.104	11.6	8.0	3.6	6.0	13.9	5.7	34.4
31	GC4100-6-5	4100 m; Line 6	Qtz in sericite-quartz zone in rhyolitic tuff	Quartz	Quartz-Sericite	167-449 (300)	0.274	4.1	5.2	-1.1	5.2	14.1	7.2	7.8
32	GC4100-2-9	4100 m; Line 2	Qtz in sericite-quartz zone in rhyolitic tuff	Quartz	Quartz-Sericite	204-278 (278)	0.051	2.8	5.7	-2.9	5.7	13.3	5.6	32.8
33	GC4100-2-10	4100 m; Line 2	Qtz in sericite-quartz zone in rhyolitic tuff	Quartz	Quartz-Sericite	236	0.070	4.1	6.5	-2.4	6.5	13.8	4.2	50.0
34	GC4100-5-6	4100 m; Line 5	Qtz in sericite-quartz zone in rhyolitic tuff	Quartz	Quartz-Sericite	196-354 (280)	0.404	2.0	3.4	-1.4	3.4	14.7	7.1	9.2
35	GC4100-5-9*	4100 m; Line 5	Qtz veins in altered rhyolite	Quartz	Quartz	290	0.661	17.1	12.6	4.5	10.5	13.6	6.3	20.4
36	GC4100-5-11	4100 m; Line 5	Qtz in sericite-quartz zone in rhyolitic tuff	Quartz	Quartz-Sericite	162-359 (290)	0.029	2.6	8.6	-6	8.6	13.4	6.2	23.7
37	GC4100-9-24	4100 m; Line 9	Qtz veins in altered rhyolite	Quartz	Quartz	215	0.835	20.1	—	—	16.2	14.6	3.9	53.5
38	GC4100-11-6	4100m; Line11	Qtz veins in deformed mudstone	Quartz	Quartz	245	0.679	17.1	—	—	12.1	14.3	5.2	39.6
39	GC4100-11-7	4100m; Line11	Qtz veins in massive ore	Quartz	Quartz	171-308 (270)	0.275	6.9	—	—	1.2	13.7	5.7	0.0
40	GC4160-0-1*	4160 m; Line 0	Qtz veins in altered rhyolite	Quartz	Quartz	240	0.172	14.7	8.6	6.1	9.9	12.7	3.3	60.9
41	GC4160-0-3	4160 m; Line 0	Sulfide-Qtz vein within rhyolitic tuff	Quartz	Quartz	240	0.469	13.7	—	—	8.9	13.9	4.5	45.1
42	GC4160-0-4	4160 m; Line 0	Pyrite-quartz bends within rhyolitic tuff	Quartz	Quartz	184-346 (250)	0.170	6.0	—	—	0.9	13.6	4.7	0.0
43	GC4160-0-5	4160 m; Line 0	Qtz vein and pyrite-quartz bends within rhyolitic tuff	Quartz	Quartz	240	0.156	4.1	—	—	-0.7	13.9	4.5	0.0
44	GC4160-3-2	4160 m; Line 3	Qtz veins in altered rhyolite	Quartz	Quartz	185-266 (260)	0.116	15.1	9.0	6.1	9.6	13.3	4.9	38.8

45	GC4160-3-4	4160 m; Line 3	Wide Qtz vein in altered rhyolite	Quartz	Quartz	215	1.197	18.4	—	—	14.5	14.0	3.3	60.4
46	GC4160-3-5	4160 m; Line 3	Qtz veins in altered rhyolite	Quartz	Quartz	155-282 (215)	0.140	10.3	6.6	3.7	4.8	13.7	5.3	33.4
47	GC4100-2-11	4100 m; Line 2	Qtz in sericite-quartz zone in rhyolitic tuff	Quartz	Quartz-Sericite	217-347 (280)	1.751	2.0	8.7	-6.7	8.7	14.2	6.6	15.9
**UMO with associated exhalites**														
48	GC1225	4050 m; Line 2	Qtz breccia in massive ore	Quartz	Quartz	175	0.306	18.1	—	—	15.9	14.2	0.9	94.3
49	GC4100-6-10	4100m; Line 6	Qtz lens on the top of rhyolitic tuff	Quartz	Quartz	175	0.439	20.7	—	—	18.4	15.5	2.2	76.4
50	GC4100-6-11	4100m; Line 6	Qtz breccia in massive ore	Quartz	Quartz	175	0.141	14.4	—	—	12.1	15.7	2.4	71.2
51	GC4100-0-1	4100m; Line 0	Qtz breccia in massive ore	Quartz	Quartz	110-200 (175)	0.054	13.2	—	—	10.9	15.4	2.1	73.2
52	GC4100-9-26*	4100m; Line 9	Qtz in massive ore	Quartz	Quartz	197-290 (240)	0.083	16.7	15.0	1.7	11.9	14.3	4.9	40.8
53	GC4160-3-10	4160m; Line 3	Qtz in sericite-quartz zone in massive ore	Quartz	Quartz-Sericite	236	0.771	2.4	7.4	-5	7.4	14.1	4.5	49.5
**Rhyolitic rocks in the Gacun ore district**														
	W88-5	ZK5501; 297m	Rhyolite	Rhyolite	—	—	—	1.2	—	—	—	—	—	—
	X30-1	ZK3904; 374m	Rhyolite	Rhyolite	—	—	—	1.0	—	—	—	—	—	—
	X31-2	Zk1901;183m	Rhyolite	Rhyolite	—	—	—	2.3	—	—	—	—	—	—
	GC4100-9-1	4100 m; Line 9	Silicified rhyolite	Rhyolite	—	—	—	1.2	—	—	—	—	—	—
	GC4100-9-11	4100 m; Line 9	Silicified rhyolite	Rhyolite	—	—	—	3.4	—	—	—	—	—	—
	GC4100-9-14	4100 m; Line 9	Altered rhyolite	Rhyolite	—	—	—	3.4	—	—	—	—	—	—
	GC4100-9-18	4100 m; Line 9	Altered rhyolite	Rhyolite	—	—	—	4.3	—	—	—	—	—	—
	GC4100-9-21	4100 m; Line 9	Altered rhyolite	Rhyolite	—	—	—	8.8	—	—	—	—	—	—
	GC4100-9-25	4100 m; Line 9	Altered rhyolite	Rhyolite	—	—	—	6.2	—	—	—	—	—	—

Note: δ^7Li_{fluid} was calculated using a quation ($\triangle\delta^7Li_{Quartz-fluid}$=-8.9382×（1000/T）+22.22) and homogeneous temperatures of fluid inclusions in Gacun deposit

$\delta^{18}O_{fluid}$ was calculated using a quation ($10^3ln\alpha_{石英-水}=3.34\times10^6/T^2-3.31$; Matsuhisa et al., 1979[13]) and homogeneous temperatures of fluid inclusions in Gacun deposit

$X_{seawater,}$ here, refers contribution rate (%) of seatwater to the ore-forming fluids at Gacun, which was calculated by δ^7Li_{mix}=（$\delta^7Li_{seawater}C_{seawater}X_{seawater}+\delta^7Li_{magmatic\ fluid}C_{magmatic\ fluid}X_{magmatic\ fluid}$）/($C_{seawater}X_{seawater}+C_{magmatic\ fluid}X_{magmatic\ fluid}$) (see Appendix II　Modeling calculation**)**

"—" here refers no determinable or less than determinable limit

"*" represents 8 quartz samples with secondary inclusions

Table S2　Li isotopic compositions of primary fluid inclusions and their host quartz at Gacun

**Sample number**	**Location**	**Mineralized zone**	**Homogeneous Temperature （℃）**	**1000/T（K）**	**δ^7Li_{quartz}(‰）**	**$\delta^7Li_{fluid-inclusions}$（‰）**	**$\triangle\delta^7Li_{Quartz-fluid}$（‰）**
GC4100-9-11	4100m; Line 9; H213	LSO	340	1.63	13.6	6.0	7.6
GC4100-2-3	4100m; Line 2; H124	LSO	327	1.67	21.0	13.8	7.2
GC4100-5-1	4100m; Line 5; H123	LSO	215	2.05	14.2	10.1	4.1
GC4100-9-12	4100m; Line 9; H195	LSO	185	2.18	12.6	9.8	2.8
GC4160-4-1	4160m; Line 4; H162	LSO	290	1.78	10.6	4.5	6.1
GC4100-6-3	4100m; Line 6; H50	MSO	210	2.07	11.6	8.0	3.6
GC4160-3-2	4160m; Line 3; H66	MSO	260	1.88	15.1	9.0	6.1
GC4160-3-5	4160m; Line 3; H44	MSO	215	2.05	10.3	6.6	3.7
GC4100-5-14	4100m; Line 5; H29	UMO	175	2.23	6.7	4.6	2.1

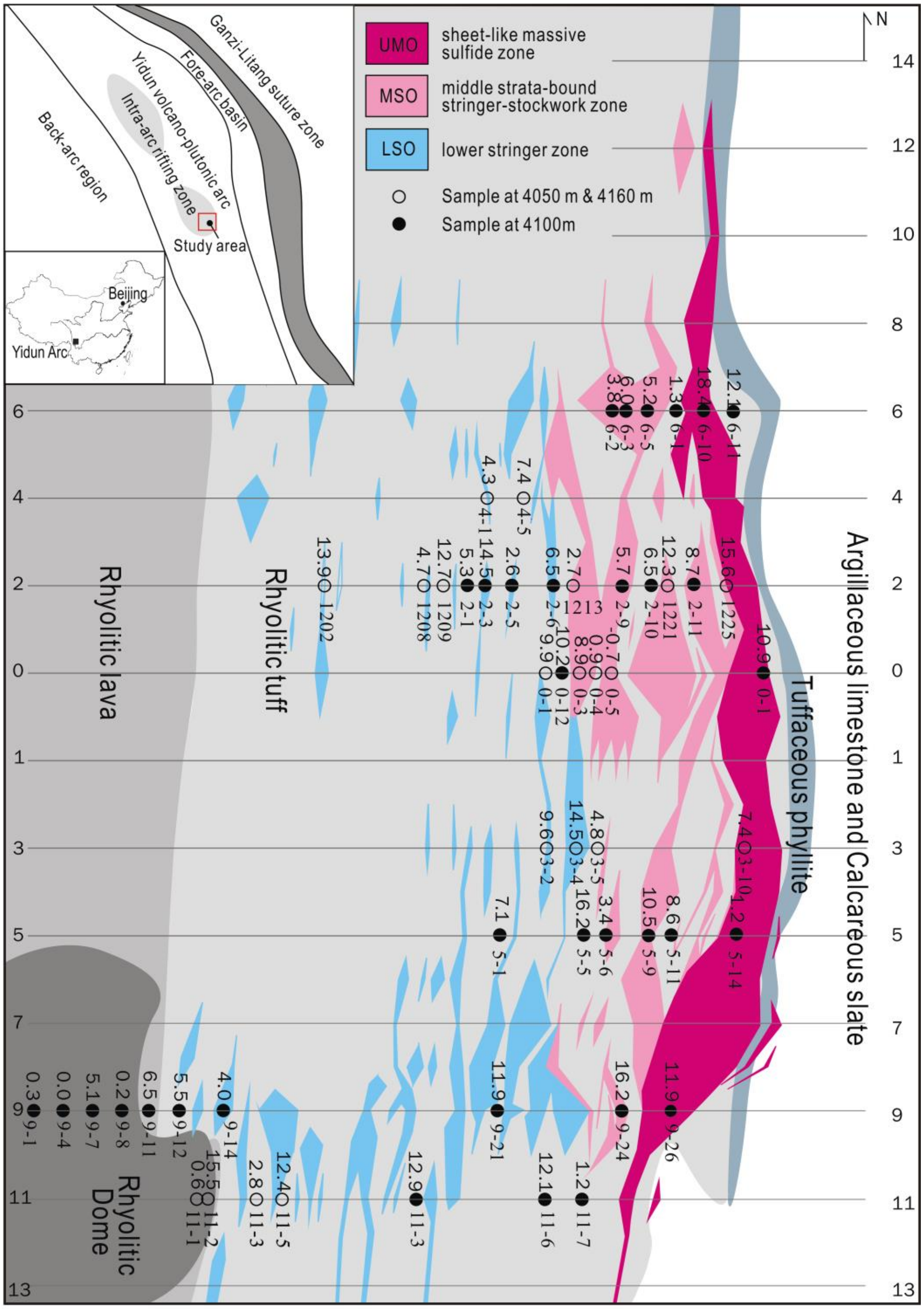

Fig.S1

Fig. S1 Point data for each sample are δ^7Li values measured for the fluid inclusions in quartz-sericite samples and calculated for the trapped fluids in pure-quartz samples. All data

from Table S1.
